# Aloe Vera as an Adjunct in Endodontic Irrigation: Impact on Dentin Bond Strength and Cytotoxicity

**DOI:** 10.3390/ma18122874

**Published:** 2025-06-18

**Authors:** Lucas David Galvani, Ester Alves Ferreira Bordini, Diana Gabriela Soares, Joatan Lucas de Sousa Gomes Costa, José Rodolfo Verbicário, Fernando Pozzi Semeghini Guastaldi, Milton Carlos Kuga, Luís Geraldo Vaz

**Affiliations:** 1 Department of Dental Materials and Prosthodontics, School of Dentistry at Araraquara, São Paulo State University (UNESP), Araraquara 14801-395, SP, Brazil; geraldo.vaz@unesp.br; 2 Department of Restorative Dentistry, Estácio University Center, Ribeirão Preto 14096-160, SP, Brazil; 3 Department of Dental Materials and Prosthesis, School of Dentistry of Ribeirão Preto, University of São Paulo (USP), Ribeirão Preto 14040-904, SP, Brazil; esterbordini@usp.br; 4 Department of Dental Materials and Prosthesis, School of Dentistry of Bauru, University of São Paulo (USP), Bauru 17012-901, SP, Brazil; dianasoares@fob.usp.br; 5 Department of Restorative Dentistry, School of Dentistry, Federal University of Minas Gerais (UFMG), Belo Horizonte 31270-901, MG, Brazil; joatan_costa@hotmail.com; 6 Department of Restorative Dentistry, School of Dentistry, São Paulo State University (UNESP), Araraquara 14801-395, SP, Brazil; rodolfoverbicario@hotmail.com (J.R.V.); milton.kuga@unesp.br (M.C.K.); 7 Skeletal Biology Research Center, Department of Oral and Maxillofacial Surgery, Massachusetts General Hospital, Harvard School of Dental Medicine, Boston, MA 02114, USA; fguastaldi@mgh.harvard.edu

**Keywords:** adhesion, fiber post, aloe vera, bond strength, biocompatible materials, cell survival

## Abstract

This study evaluated the effects of mechanical agitation of Aloe vera Barbadensis Miller solution at different concentrations using passive ultrasonic irrigation (PUI), XP Endo Finisher (XPF), XP Clean (XPC), and Easy Clean (ECL), compared to conventional endodontic irrigation (CIE), on bond strength and adhesive failure patterns in the cervical, middle, and apical thirds of the root canal. Aloe vera solutions at 1%, 3%, and 5% were tested to reverse collagen fiber collapse induced by hypochlorous acid, a free radical released by 2.5% sodium hypochlorite, which impairs dentin hybridization and the light curing of resin cement. Fiberglass posts were cemented using an etch-and-rinse adhesive system (Ambar; FGM) and conventional dual resin cement (Allcem Core) in root dentin across all thirds. Human teeth underwent chemical–mechanical preparation, and the Aloe vera solution was agitated using the CIE, PUI, XPF, XPC, or ECL protocols. Slices from each root third were evaluated under a stereomicroscope at 10× magnification and subjected to the push-out test. Cytotoxicity was assessed by applying various Aloe vera concentrations to stem cells from the apical papilla (SCAPs) for 24 h, followed by analysis of cell metabolism (Alamar Blue), viability (Live/Dead), and proliferation (F-actin). Aloe vera demonstrated significant biological activity and enhanced bond strength, particularly at 3% and 5%, irrespective of the agitation method or root third. Thus, it can be concluded that using Aloe vera solution is an alternative for pre-treatment before the cementation of fiberglass posts with conventional dual-cure resin cement in endodontically treated dentin.

## 1. Introduction

The success of endodontic treatment relies on an effective chemomechanical preparation capable of eliminating bacteria, microorganisms, and residual pulp tissue from the root canals [1]. Following this phase, a three-dimensional obturation of the canals is essential to fill the spaces previously occupied by the pulp tissue. Once these steps are properly completed, a favorable environment is established for prosthetic dental rehabilitation.

The formation of the hybrid layer is crucial for enhancing the bond strength between radicular dentin and the restorative material. This layer involves an interaction between the adhesive system polymer and dentinal collagen [2]. To achieve an optimal hybrid layer, the dentin surface must be conditioned with either exogenous acids or self-etching monomers. These acids expose collagen fibers and keep the dentinal tubules open, facilitating the penetration of the primer and, consequently, the adhesive [3].

Teeth undergoing endodontic therapy come into direct contact with sodium hypochlorite, the irrigant of choice due to its antimicrobial properties, organic tissue dissolving action, lubricating effect during chemomechanical preparation, and its ability to penetrate and remove microorganisms and dentinal debris resulting from instrumentation [4]. However, sodium hypochlorite exhibits oxidative activity, driven by the release of singlet oxygen derived from the ionic dissociation of hypochlorous acid [5]. The free radicals generated in this process can reduce the degree of conversion of resin-based compounds and occupy the intratubular space and/or the interfibrillar region of dentinal collagen, ultimately compromising adhesion to the dentin substrate [6,7].

Dentin degradation occurs due to the collapse of collagen fibers, interfering with the formation of the hybrid layer and contributing to adhesive failures in the remaining dental structure. Matrix metalloproteinases (MMPs-2, 3, 8, 9, and 20) are the primary enzymes responsible for collagen degradation in human teeth [8,9]. Antioxidant agents can mitigate the deleterious effects of free radicals at the adhesive interface, although their action is localized, with limited diffusion through dentin [10,11]. Final irrigation with chemically inert substances, combined with mechanical activation, can minimize the residual effects of sodium hypochlorite on the dentin surface and interior, restoring optimal conditions for hybridization with etch-and-rinse adhesive systems and conventional resin cement [5].

Within this context, Barbadensis Miller (Aloe vera) is a succulent plant resembling a cactus from the Aloe genus, native to Africa, with medicinal and hydrating properties commonly used to treat burns and skin diseases [12,13]. In recent years, Aloe vera has been investigated in the field of endodontics to assess its potential to inhibit MMP activity and its antimicrobial effects on persistent periodontal pathogens [8,14]. Despite the relevance of Aloe vera in the biological modulation of tissues, few studies in the literature have explored its potential effects on the dentin surface, particularly its possible hydrating properties in reversing collagen fiber collapse. This property could enhance dentin hybridization, which is often compromised by the action of hypochlorous acid.

Another fundamental aspect is the evaluation of the cytotoxicity of Aloe vera on human apical papilla cells (SCAPs), considering not only its direct contact with apical tissues but also its interaction with the periodontium. This analysis is crucial given the anatomical complexity of root canal systems. Furthermore, the high prevalence of lateral canals—identified in 65% of incisors, 89% of premolars, and 84% of molars—and interradicular furcal canals in 4% of molars underscore the significance of this investigation [12].

Thus, the present study aimed to evaluate the effects of conventional final irrigation protocols using Aloe vera solution at different concentrations on promoting the reversal of collagen fiber collapse induced by hypochlorous acid and to assess the cytocompatibility of this solution with human apical papilla cells (SCAPs). Additionally, this study proposed activating the Aloe vera solution using different irrigation protocols with XP Endo Finisher, XP Clean, or passive ultrasonic irrigation (PUI) following neutralization with 2.5% sodium hypochlorite solution. Finally, the study evaluated the effect of the employed solutions, with or without activation, on bond strength and the adhesive failure pattern of fiber post cementation, using the Ambar adhesive system (Ambar; FGM, Joinville, SC, Brazil) and conventional dual-cure resin cement (Allcem Core; FGM, Joinville, SC, Brazil) in different thirds of the root dentin.

The null hypothesis 1 (H01) considered that the solution containing Aloe vera is not capable of reversing collagen fiber collapse, thereby not improving the formation of the hybrid layer. As null hypothesis 2 (H02), we assumed that the mechanical activation of the Aloe vera solution using different inserts does not affect the bond strength of conventional resin cement. Conversely, the alternative hypothesis (H1) posits that the Aloe vera solution has a promising impact on reversing collagen fiber collapse and that its association with activation inserts will provide benefits in the formation of an adequate hybrid layer, enhancing the cementation pattern within root canals. Furthermore, we aim to demonstrate that Aloe vera does not exhibit cytotoxicity when in contact with SCAPs cultured with different solution concentrations.

## 2. Materials and Methods

### 2.1. Preparation of the Solution for Biological Analyses

The Aloe vera Barbadensis Miller powder was purchased from the compounding pharmacy Engenharia das Essências Comercial LTDA, registered under CNPJ 36.437.409/0001-17 in São Paulo, SP, Brazil.

A quantity of 1 g of Aloe vera powder was incorporated into 10 mL (*w*/*v*) of dimethyl sulfoxide (DMSO; Sigma-Aldrich, Saint Louis, MO, USA), previously stored in a Falcon tube. The mixture was then vortexed until a homogeneous solution was obtained. From this stock solution, working solutions were prepared at concentrations of 0.25%, 0.50%, 0.75%, 1%, 2%, 3%, and 5%, which were diluted in complete α-MEM (Minimum Essential Medium Eagle Alpha), supplemented with 10% fetal bovine serum (FBS), L-glutamine, and 1% penicillin–streptomycin (Gibco^®^, Invitrogen, Carlsbad, CA, USA) for biological analyses.

To assess adhesion resistance in endodontically treated root dentin, the Aloe vera solution was prepared following the same criteria mentioned above, except for using deionized water as the solvent at concentrations of 1%, 3%, and 5%.

### 2.2. In Vitro Experimental Design

SCAPs were cultured until 80% confluence and then trypsinized and seeded (2 × 10^3^ cells/well) in 96-well plates (Corning^®^, Corning, NY, USA) containing complete α-MEM. The cells were incubated for 24 h to allow for cell growth. After this period, the culture medium was replaced with α-MEM containing 10% fetal bovine serum (FBS), supplemented or not with Aloe vera solutions at concentrations of 1%, 3%, and 5%. The solutions were applied separately and remained in contact with the cells for 24 h, followed by assessments of cellular metabolism (Alamar Blue), cell viability (Live/Dead), and cell adhesion/spreading (F-actin).

### 2.3. Cell Metabolism (Alamar Blue)

After one day of culture, SCAPs were evaluated for cell proliferation using the Alamar Blue reagent. A solution of α-MEM without FBS containing 10% Alamar Blue reagent (Life-Technologies, Carlsbad, CA, USA) in a 10:1 ratio was prepared and maintained in contact with the cells for 4 h at 37 °C and 5% CO_2_. After this period, the supernatant was transferred to 96-well plates (Corning^®^), and fluorescence was measured (540 nm excitation–590 nm emission; Synergy H1, Biotek, Winooski, VT, USA). The mean fluorescence value obtained for the negative control group (cells cultured in a complete medium without Aloe vera solution) was considered 100% cell proliferation, and the proliferation percentage of the experimental groups was calculated based on this parameter (*n* = 6).

### 2.4. Cell Viability (Live/Dead)

After 24 h of culture, the cytocompatibility of the different concentrations of Aloe vera solutions was assessed using a Live/Dead assay (*n* = 3). For this purpose, the cells seeded in the plate were incubated with culture medium without FBS, supplemented with Calcein AM and Ethidium Homodimer-1 (Live/Dead Cell Viability/Cytotoxicity Kit; Invitrogen, San Francisco, CA, USA) at a 1:1000 concentration for 15 min. Subsequently, each well was washed three times with 1× PBS, and the cell surface was analyzed under a fluorescence microscope (EVOS FLoid Cell Imaging Station; Invitrogen, Waltham, MA, USA).

### 2.5. Cell Adhesion and Spreading (F-Actin)

Cell adhesion and spreading on the well surfaces were assessed after one day of culture with different concentrations of Aloe vera solutions, through the analysis of F-actin filaments. For this purpose, each well (*n* = 3) was initially washed with PBS, and the cells were then fixed with 4% paraformaldehyde (PFA; Sigma-Aldrich) for 15 min. After this period, the Alexa Fluor Phalloidin 555 fluorescent probe was prepared in bovine serum albumin (BSA) at a 1:50 ratio (Life Technologies, Carlsbad, CA, USA) and applied to the cells for 30 min. Each well was washed with PBS to remove excess dye, and nuclear counterstaining was performed using the DAPI mounting medium (ProLong, Thermo Fisher Scientific, Waltham, MA, USA). The analyses were conducted using a fluorescence microscope (EVOS FLoid Cell Imaging Station; Invitrogen™, Waltham, MA, USA) to obtain representative sample images.

### 2.6. Sample Preparation

One hundred and sixty sound maxillary central incisors were obtained from the tooth bank of the School of Dentistry at Araraquara (FOAR-Unesp) (CAAE65219622.6.0000.5416). The teeth were immersed in a saline solution and stored under refrigeration at 4 °C until use. The roots were transversely sectioned using a diamond disc adapted to a hard tissue cutting machine (Isomet 2000; Buehler Ltd., Lake Bluff, IL, USA) and standardized to a length of 21 mm from the apical root.

Root canal treatment was performed by a single operator, following the protocol described by Aranda-Garcia et al. [15]. After the cervical sectioning of the root canal filling, the cervical access was temporarily restored with glass ionomer cement (Maxxion R; FGM, Joinville, SC, Brazil). The specimens were stored at 37 °C with 100% humidity for seven days. After removing the glass ionomer, the post space was prepared using Largo drills #1 and #2 (Dentsply Maillefer; Ballaigues, Jura-Nord Vaudois, Switzerland) and finalized with drill #2 (White Post DC; FGM, Joinville, SC, Brazil) to a depth of 18 mm from the cervical root surface.

### 2.7. Preparation and Irrigation of the Post Space

The post space was initially irrigated with 5 mL of distilled water. Subsequently, the specimens were randomly distributed and conditioned with 37% phosphoric acid for 15 s, followed by irrigation with distilled water for 30 s. After conditioning and removing the 37% phosphoric acid, the specimens were divided into four groups, namely a control group, irrigated with distilled water (*n* = 10), and three experimental groups (*n* = 50), based on different concentrations of Aloe vera solution. The experimental groups received Aloe vera (Barbadensis Miller) solutions at concentrations of 1%, 3%, and 5%. Each group was then subjected to a specific solution activation protocol before the cementation of the fiberglass post, as described below:

**Control**: The post space was irrigated with 15 mL of distilled water using an irrigation cannula (Navitip 29G; Ultradent, Indaiatuba, SP, Brazil) inserted 3 mm short of the full post space length (15 mm) for 1 min, with slight 2 mm apicocoronal movements.

**CEI**—Conventional Endodontic Irrigation: The post space was irrigated with 15 mL of Aloe vera (Barbadensis Miller) solution using an irrigation cannula (Navitip 29G; Ultradent, Indaiatuba, SP, Brazil), inserted 3 mm short of the full post space length (15 mm), for 1 min, with slight 2 mm apicocoronal movements.

**PUI**—Passive Ultrasonic Irrigation: The post space was filled with Aloe vera (Barbadensis Miller) solution and energized using an ultrasonic insert (Irrisonic E1; Helse Technology, Santa Rosa de Viterbo, SP, Brazil), adapted to an ultrasonic device (Piezon 150; Electron Medical Systems, Nyon, Switzerland), at 10% power, inserted into the post space 2 mm short of its full length (16 mm), for 20 s. This procedure was repeated for two additional cycles, totaling three activation cycles of 20 s each.

**XPF**—Activation with XP Endo Finisher (FKG Dentaire; Madrid, Spain): After removing the packaging, the blister containing the device was cooled with a pulpal sensitivity spray, following the manufacturer’s recommendation (Endo Ice; Maquira, Maringá, PR, Brazil). Simultaneously, the root canal was filled with Aloe vera solution and preheated to 37 °C. After adapting the device to an electric contra-angle handpiece (XSmart Plus; Dentsply Maillefer, Pirassununga, SP, Brazil), the insert was introduced along the entire post space length and activated at a speed of 1000 rpm with a continuous torque of 1 N for 1 min.

**XPC**—Activation with XP Clean (MKLife; Porto Alegre, RS, Brazil): The XP Clean device activation procedures followed the same protocol as the XPF group.

**ECL**—Activation with Easy Clean (Easy; Belo Horizonte, MG, Brazil): The device was previously calibrated using a gauge ruler (Maillefer, Pirassununga, SP, Brazil), ensuring that D0 corresponded to a #50 LK instrument. The post space was filled with Aloe vera solution, and after inserting the instrument to the full post space length, it was activated using an electric device (XSmart Plus; Dentsply Maillefer, Pirassununga, SP, Brazil), pre-programmed for rotational movement at a speed of 1000 rpm with 1 N of torque, in three cycles of 20 s each.

After each irrigation protocol, the post space was aspirated and dried with absorbent paper points before the cementation of the fiberglass post.

### 2.8. Bond Strength

After preparing each group, the dentin was gently dried using absorbent paper points (Maillefer, Dentsply Sirona, York, PA, USA). The Ambar adhesive system (Ambar; FGM, Joinville, SC, BR) was applied to the post space and light-cured for 20 s. The external surface of the fiber post (White Post DC #1; FGM, Joinville, SC, Brazil) was cleaned with 95% alcohol and etched with 37% phosphoric acid (Condac 37; FGM, Joinville, SC, Brazil) for 1 min. Subsequently, a silane coupling agent (Prosil; FGM, Joinville, SC, Brazil) and the Ambar adhesive (Ambar; FGM, Joinville, SC, Brazil) were applied and light-cured for 60 s using a Valo curing unit (Ultradent, Salt Lake, UT, USA). The dual-cure resin cement (Allcem Core; FGM, Joinville, SC, Brazil) was prepared according to the manufacturer’s instructions and inserted into the post space. The fiberglass post was then positioned and light-cured for 40 s on each side of the specimen. The specimens were stored in mineral oil (Nujol; Matecorp Farmasa, São Paulo, SP, Brazil) at 37 °C for six months.

### 2.9. Push-Out Test

After six months of storage, the roots were vertically embedded in a polyvinyl chloride matrix. These matrices were filled with polyester resin (Maxi Rubber, Diadema, SP, Brazil) up to a height of 16 mm, leaving 1 mm of the cervical third exposed [5]. After 24 h, the specimens were removed from the matrices and sectioned transversely using a diamond saw mounted on a water-cooled precision cutting machine (Isomet 1000, Buehler Ltd., Lake Bluff, IL, USA). Three slices, each 2 mm ± 1 mm thick, were obtained from the post space’s apical, middle, and cervical thirds. Cervical, middle, and apical sections were obtained at 3, 9, and 15 mm from the cervical root surface. Irregularities were removed using #1200 grit silicon carbide sandpaper (Norton, São Paulo, SP, Brazil).

The push-out test was performed using an electromechanical testing machine (EMIC, São José dos Pinhais, PR, Brazil) with a 5 kN load cell at 0.5 mm/min crosshead speed. To displace the fiber post/cementation system, a specific notched crosshead was used for axial displacement in each third: 1.2 mm for the cervical third, 0.9 mm for the middle third, and 0.5 mm for the apical third. The maximum force required to displace the post system was recorded in Newtons (N) to assess the bond strength values, as described by Magro et al. (2014) [16].

### 2.10. Incidence of Adhesive Failure Mode

After the push-out bond strength test, each specimen was polished using #600 and #1200 grit waterproof sandpaper (Norton, Lorena, SP, Brazil). The specimens were then washed with distilled water, and the cervical surface was polished with 30 μm aluminum oxide (Arotec, São Paulo, SP, Brazil). Next, the specimens were immersed in distilled water and subjected to ultrasonic agitation (Cristófoli, Campo Mourão, PR, USA) for 10 min. The specimens were then air-dried and horizontally mounted on a glass slide.

**Figure 1:**
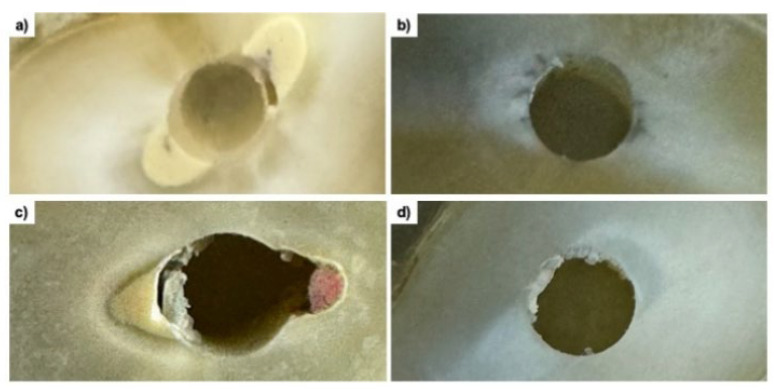
Failure mode: (**a**) type 1 (adhesive 1)—failure between the fiber post and the cement; (**b**) type 2 (adhesive 2)—failure between the dentin and the cement; (**c**) type 3 (cohesive)—failure within the cement; (**d**) type 4 (mixed)—a combination of different failure types.

To determine the failure modes in each third of the post space, the specimens were examined under a stereomicroscope at 10× magnification and classified according to Ramos et al. [5], as illustrated in Figure 1.

### 2.11. Statistical Analysis

The biological analyses and bond strength test data were initially subjected to the Shapiro–Wilk test (*p* > 0.05). Subsequently, the biological analyses were evaluated using the Kruskal–Wallis test, followed by Dunn’s post hoc test. A one-way ANOVA was performed for the bond strength analysis, followed by Tukey’s post hoc test. All analyses were conducted with a significance level of 5%. The GraphPad Prism 9 software (San Diego, CA, USA) was used for data analysis. Failure mode was qualitatively assessed.

## 3. Results

### 3.1. Cell Metabolism (Alamar Blue)

**Figure 2:**
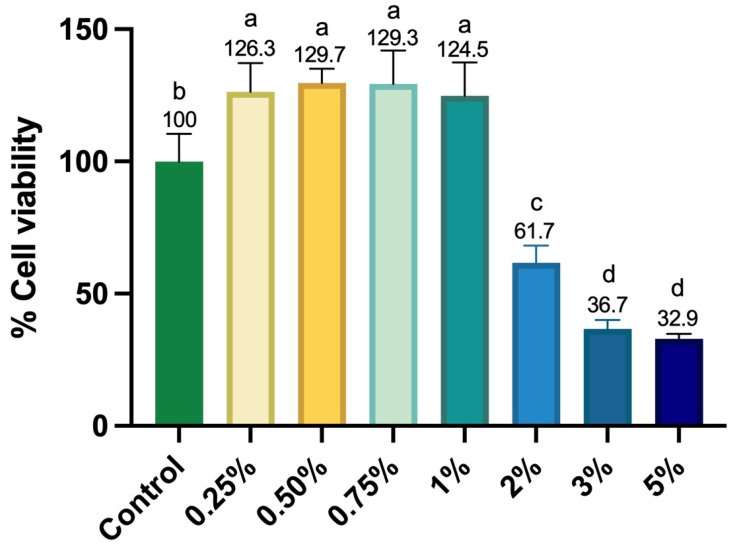
Alamar Blue assay. Boxplot graph represents the experimental and control groups’ medians, 1st, and 3rd quartiles. Different letters indicate a statistically significant difference between groups (Kruskal–Wallis; Dunn’s, *p* < 0.05, *n* = 6).

After culturing human apical papilla stem cells (SCAPs) with different concentrations of Aloe vera solution (0.25%, 0.5%, 0.75%, 1%, 2%, 3%, and 5%), a significant increase in cellular metabolism was observed at concentrations of 0.25%, 0.5%, 0.75%, and 1%. This increase averaged 27.5% compared to the control group, demonstrating that these solutions were biocompatible and enhanced SCAP metabolism within just 24 h (Figure 2). Conversely, the 2% concentration reduced cellular metabolism by 38.6%, while 3% and 5% concentrations led to cell death exceeding 60% (Figure 1).

The increased cell viability observed with the lower concentration of the Aloe vera solution may be attributed to the concentration-dependent behavior of its bioactive constituents. At subcytotoxic levels, compounds such as acemannan, polysaccharides, and antioxidant vitamins (notably C and E) can exert cytoprotective and proliferative effects by reducing intracellular oxidative stress, preserving mitochondrial integrity, and modulating key prosurvival signaling pathways, such as PI3K/Akt and MAPK/ERK [17,18,19]. This favorable cellular response likely reflects an optimal biological window in which Aloe vera supports cellular homeostasis and metabolic activity. Conversely, at higher concentrations, the accumulation of anthraquinones and other secondary metabolites may disrupt redox balance, promote oxidative damage, and compromise membrane integrity, resulting in reduced viability [20,21].

### 3.2. Cell Viability (Live/Dead)

**Figure 3:**
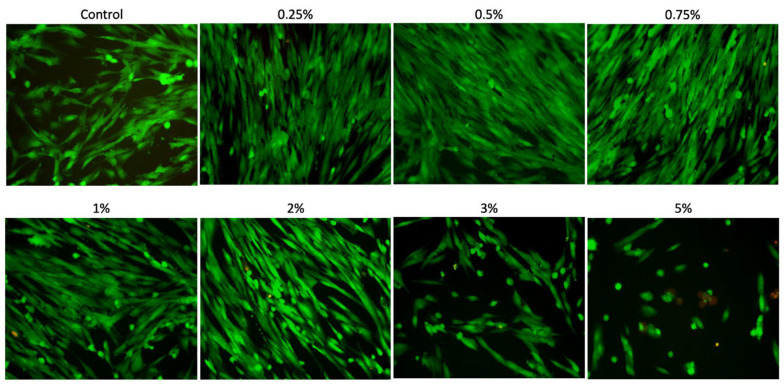
Live/Dead assay. Representative images (20×; 100 μm) of SCAPs cultured with different concentrations of Aloe vera (0.25%, 0.5%, 0.75%, 1%, 2%, 3%, and 5%) for 24 h. Green staining = viable cells; red staining = dead cells.

The Live/Dead assay (Figure 3) showed that the cells remained viable after 24 h of treatment with Aloe vera solutions at concentrations of 0.25%, 0.5%, 0.75%, 1%, and 2%, displaying a high number of live cells (stained green) and few or no dead cells (stained red). Furthermore, these concentrations allowed the cells to maintain a morphology similar to that of the control group. Higher concentrations (3% and 5%) exhibited fewer adhered cells, reducing cell viability. Additionally, the 5% concentration induced the cells to organize into clusters, with dead cells (red staining).

### 3.3. Cell Adhesion/Spreading (F-Actin)

**Figure 4:**
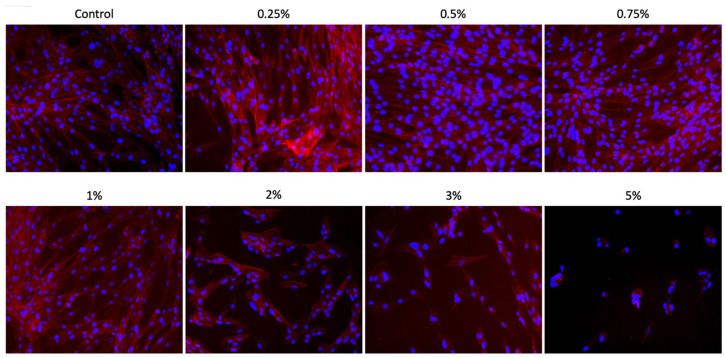
Cell adhesion/spreading assay. Representative images (20× 100 μm) of SCAPs cultured with different concentrations of Aloe vera (0.25%, 0.5%, 0.75%, 1%, 2%, 3%, and 5%) for 24 h. Blue staining = nuclei; red staining = F-actin filaments.

Aloe vera solutions at concentrations of 0.25%, 0.5%, 0.75%, and 1% demonstrated excellent biocompatibility, allowing cells to spread across the entire plate surface and exhibit an elongated cytoskeleton characteristic of SCAPs, with morphology similar to the control group (Figure 3). Due to their cytotoxicity, concentrations of 2%, 3%, and 5% resulted in a reduced number of adhered cells, which presented a rounded morphology, particularly at 5%. The results from the F-actin assay corroborate those obtained from the Alamar Blue metabolic assay (Figure 4).

### 3.4. Push-Out Bond Strength Test

**Figure 5:**
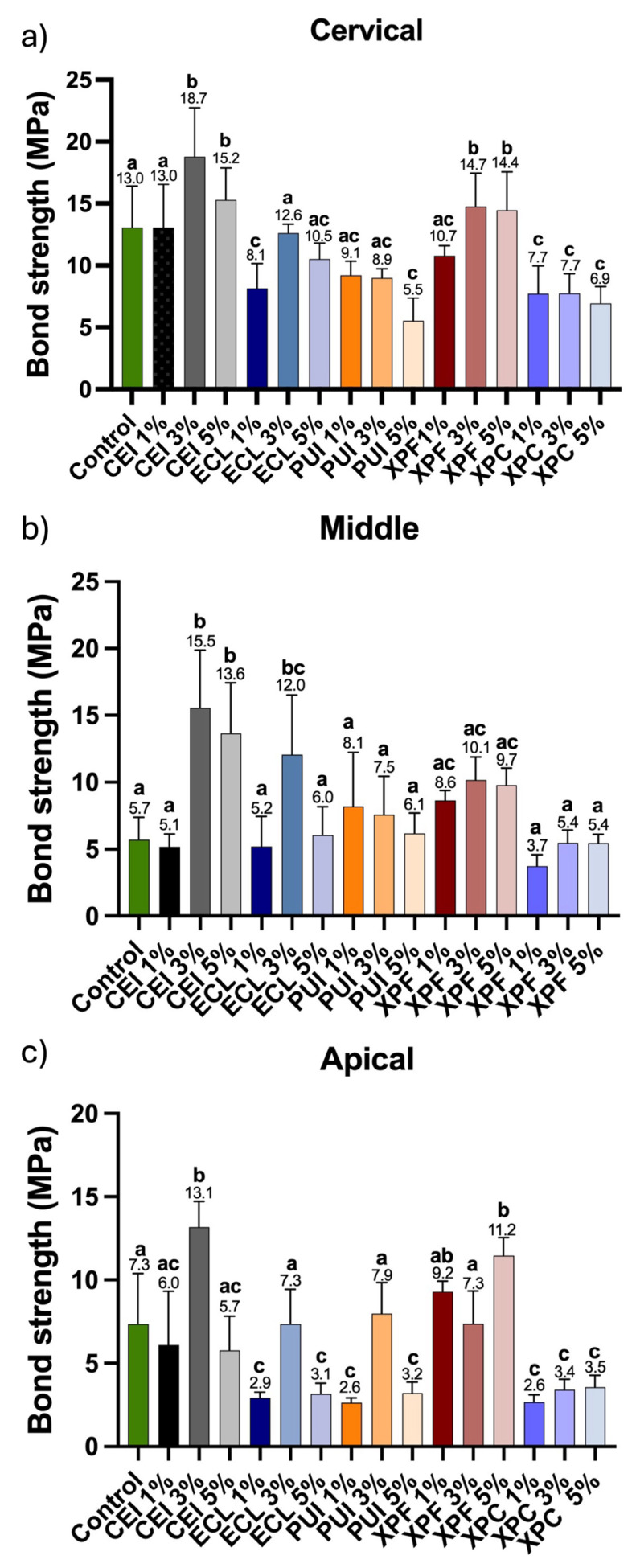
Representative graph of the maximum tensile strength (MPa) in the (**a**) cervical, (**b**) middle, and (**c**) apical third of the root. Different letters indicate statistically significant differences between groups (one-way ANOVA; Tukey’s post hoc test, *p* < 0.05; *n* = 10/group).

In the cervical third of the root, higher maximum tensile strength values were observed in the conventional irrigation (CEI) and XP Endo (XPF) groups treated with Aloe vera at 3% and 5% concentrations. The experimental groups Easy Clean (ECL 3%), PUI (1% and 3%), XP Endo (XPF 1%), and XP Clean (XPC 5%) exhibited tensile strength values comparable to the control group, ranging from 9.1 to 23.0 MPa. In contrast, the ECL 1%, PUI 5%, and XPC at concentrations of 1%, 3%, and 5% showed the lowest maximum tensile strength, with values ranging from 5.5 to 8.1 MPa (Figure 5a).

In the middle third of the root, the groups that exhibited the maximum tensile strength (MPa) were the CEI 3%, CEI 5%, and ECL 3% groups. The other experimental groups showed statistically similar values to the control group, presenting lower maximum tensile strength. The lowest value was observed for the XPC insert at 1% concentration, with a maximum tensile strength of 3.7 MPa (Figure 5b).

In the apical third of the root, the groups that exhibited the highest maximum tensile strength (MPa) were CEI 3% (13.1 MPa), XPF 1% (9.0 MPa), and XPF 5% (12.2 MPa). The control group (7.3 MPa) showed similar results to CEI 1% (6.0 MPa), CEI 5% (5.7 MPa), ECL 3% (7.3 MPa), PUI 3% (7.9 MPa), and XPF 3% (7.3 MPa). The remaining experimental groups demonstrated the lowest tensile strength values, with no statistically significant differences ranging from 2.6 to 3.5 MPa (Figure 5c).

### 3.5. Failure Mode Incidence

**Figure 6:**
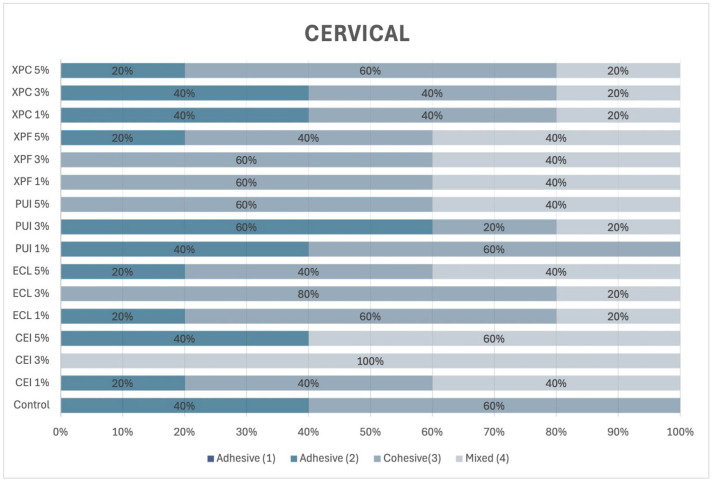
Incidence of failure mode in percentage in the cervical third of the root.

In the cervical third of the root (Figure 6), cohesive failures were predominant regardless of the group or the concentration of Aloe vera. Adhesive failure was observed exclusively in the control group.

**Figure 7:**
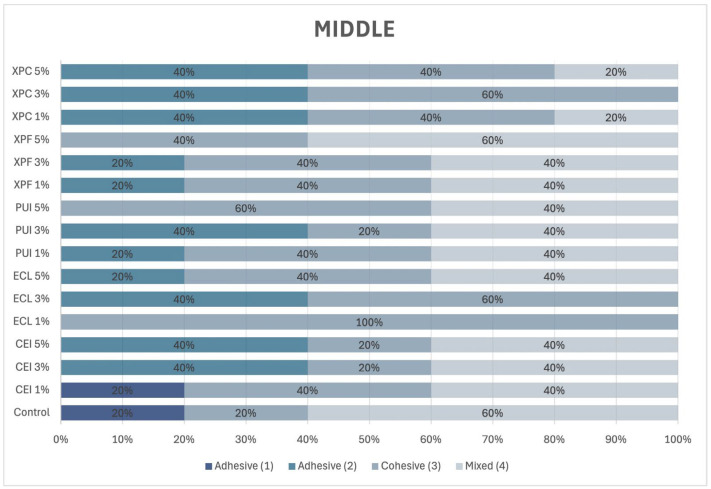
Percentage incidence of failure mode in the middle third of the root.

In the middle third of the root (Figure 7), a predominance of mixed failure was observed regardless of the concentration of Aloe vera. A similar incidence of adhesive and cohesive failures was observed in the XP Endo Finisher group.

**Figure 8:**
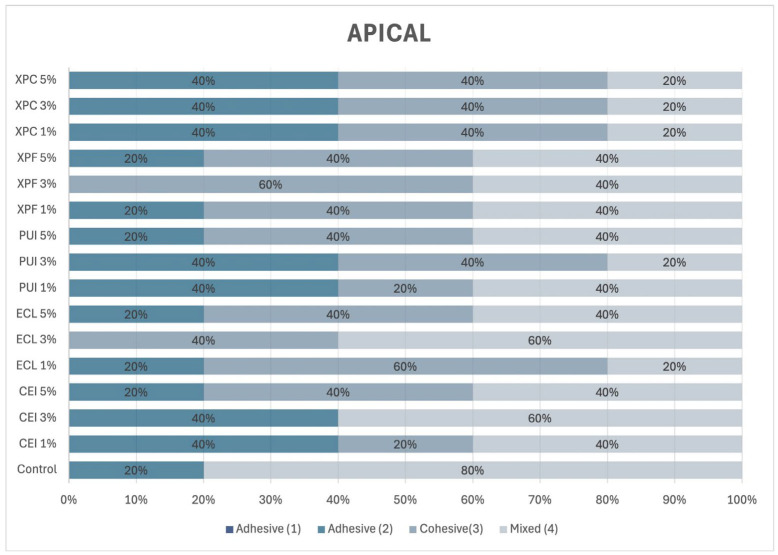
Failure mode incidence (%) in the apical third of the root.

In the apical third of the root (Figure 8), a predominance of mixed failure was observed regardless of the concentration of Aloe vera. Only in the XPF group, at all the tested concentrations, and in the PUI 3% and ECL 3% groups, were adhesive and cohesive failures observed with comparable frequency.

## 4. Discussion

Hypothesis 1 (H01), which proposed that the solution containing Aloe vera would not be able to reverse collagen fiber collapse and would not enhance hybrid layer formation, was rejected. It was observed that the solution’s 3% and 5% concentrations resulted in higher bond strength between the root dentin surface and the fiber post cementation system when compared to the control group, which used distilled water. Hypothesis 2 was confirmed, demonstrating that using different irrigant activation devices did not influence the effect of the Aloe vera solution. The results obtained for the conventional irrigation (CEI) group were comparable to those of the XP Endo Finisher (XPF) group and even superior to those of the other groups. The alternative hypothesis, which suggested that activation devices would enhance the effect of the solution on root dentin compared to syringe irrigation, was therefore rejected.

The use of plants for alternative treatments has been extensively investigated in the fields of healthcare and medicine due to their high potential for hydration, regeneration, and healing of various pathologies. In the dental context, Kaual et al. [17] evaluated the antimicrobial potential of Aloe vera on acrylic resins, observing an improvement in material hardness and antimicrobial efficacy compared to conventional acrylic resins.

In the present study, we evaluated the potential of Aloe vera at different concentrations on root dentin in the cervical, middle, and apical thirds. A significant improvement in the bond strength between the root surface and the fiber post cemented with dual-cure resin cement was observed at concentrations of 3% and 5%, compared to the control groups and the 1% Aloe vera solution. This result was expected, given that Aloe vera is widely used in medicine as a natural healing and moisturizing agent. However, its potential for dentin rehydration, particularly following endodontic treatment, during which root dentin undergoes significant dehydration due to contact with sodium hypochlorite, has not yet been explored.

Considering this context, an innovative solution containing Aloe vera at different concentrations was formulated and directly applied to SCAPs. Stem cells from the apical papilla are undifferentiated mesenchymal cells with high potential for application in tissue regeneration processes, enabling the regeneration of tissues such as pulp, dentin, periodontal structures, and even bone tissue [22,23]. Therefore, preserving their viability in the apical region of permanent teeth is essential—even in teeth undergoing endodontic treatment—since studies have demonstrated that these cells can remain metabolically active, proliferating, and differentiating even under inflammatory conditions [22,24].

Thus, when evaluating the bioactive potential of Aloe vera solutions in contact with SCAPs, it was observed that the concentrations of 0.25%, 0.5%, 0.75%, 1%, and 2% promoted an increase in metabolism, viability, proliferation, and cell spreading when compared to the concentrations of 3% and 5%. Other studies in the literature have also demonstrated the beneficial potential of Aloe vera. In a study developed by Namazi et al. [25], it was demonstrated that human dental pulp cells, when in contact with a hydrogel containing Aloe vera, showed an increase in cell viability, in addition to exhibiting anti-inflammatory and antimicrobial effects, supporting the application of this solution within root canals in endodontic therapies. Furthermore, the study by Sholehvar et al. [26] aimed to develop a gel for tissue regeneration and demonstrated that, when in contact with DPSCs, there was an increase in cell viability and cell differentiation potential due to the nutrients present in Aloe vera, which promote cytoprotective effects on the cells.

Therefore, it is noteworthy that Aloe vera exhibits significant biological potential, which is associated with a novel characteristic evaluated in this study: the enhancement of bond strength between dual-cure resin cement and root dentin. Simultaneously, its biocompatible potential in contact with SCAPs was confirmed.

Laboratory research using extracted human teeth is widely accepted for the initial screening of dental materials, as it allows for the control of variables and facilitates reproducibility. However, the absence of the periodontal ligament, physiological temperature, and natural dentin hydration represents an important limitation of the present study, potentially leading to an overestimation of bond strength values. Additionally, the experimental design did not include thermocycling or mechanical loading protocols that better simulate the in vivo oral environment, which should be addressed in future investigations.

During sample preparation, dentin was etched with 37% phosphoric acid, followed by an initial rinse with distilled water and subsequent application of the Aloe vera solution. This sequence may have influenced the results, and future studies should evaluate the efficacy of using Aloe vera solution alone for acid removal. Although concentrations of 3% and 5% showed significant cytotoxicity in biological assays, they were included in the next experimental phase to assess their potential use in root canal irrigation. Conversely, lower concentrations, which exhibited excellent biocompatibility in vitro, may be promising for future adhesive protocols in vital teeth. Furthermore, the present study did not evaluate the long-term stability of adhesive bonds or the antimicrobial effectiveness of the tested formulations, both of which are essential for clinical applicability. Finally, the biological behavior of the Aloe vera solution, particularly its adverse effects on vital cellular processes, requires further investigation to identify the underlying mechanisms and improve the translational relevance of the material.

## 5. Conclusions

This study formulated Aloe vera solutions at different concentrations to develop a biocompatible solution with root dentin and apical and periapical tissues. After cell viability analyses, the biocompatible concentrations were selected and subjected to bond strength tests using different inserts for solution activation on root dentin. It was observed that the use of Aloe vera demonstrated active biological potential and benefits in the bond strength of dual-cure resin cement to root dentin, especially at concentrations of 3% and 5%. These effects were observed both with and without inserts for solution agitation, regardless of the root third evaluated. Therefore, it can be concluded that using Aloe vera solution is an alternative that can be employed prior to the cementation of glass fiber posts with dual-cure resin cement in endodontically treated dentin.

## Data Availability

The original contributions presented in this study are included in the article. Further inquiries can be directed to the corresponding author.
